# Recent Advances in the Development of Sulfamoyl-Based Hepatitis B Virus Nucleocapsid Assembly Modulators

**DOI:** 10.3390/v15122367

**Published:** 2023-11-30

**Authors:** Sandesha Nayak, Jayaraj Gowda, Syed Azeem Abbas, Hyejin Kim, Soo Bong Han

**Affiliations:** 1 Therapeutics and Biotechnology Division, Korea Research Institute of Chemical Technology, Daejeon 34114, Republic of Korea; 2 Department of Medicinal Chemistry and Pharmacology, University of Science & Technology, Daejeon 34113, Republic of Korea

**Keywords:** hepatitis B virus, HBV core protein, CAMs, cccDNA

## Abstract

Hepatitis B virus (HBV) is the primary contributor to severe liver ailments, encompassing conditions such as cirrhosis and hepatocellular carcinoma. Globally, 257 million people are affected by HBV annually and 887,000 deaths are attributed to it, representing a substantial health burden. Regrettably, none of the existing therapies for chronic hepatitis B (CHB) have achieved satisfactory clinical cure rates. This issue stems from the existence of covalently closed circular DNA (cccDNA), which is difficult to eliminate from the nucleus of infected hepatocytes. HBV genetic material is composed of partially double-stranded DNA that forms complexes with viral polymerase inside an icosahedral capsid composed of a dimeric core protein. The HBV core protein, consisting of 183 to 185 amino acids, plays integral roles in multiple essential functions within the HBV replication process. In this review, we describe the effects of sulfamoyl-based carboxamide capsid assembly modulators (CAMs) on capsid assembly, which can suppress HBV replication and disrupt the production of new cccDNA. We present research on classical, first-generation sulfamoyl benzocarboxamide CAMs, elucidating their structural composition and antiviral efficacy. Additionally, we explore newly identified sulfamoyl-based CAMs, including sulfamoyl bicyclic carboxamides, sulfamoyl aromatic heterocyclic carboxamides, sulfamoyl aliphatic heterocyclic carboxamides, cyclic sulfonamides, and non-carboxamide sulfomoyl-based CAMs. We believe that certain molecules derived from sulfamoyl groups have the potential to be developed into essential components of a well-suited combination therapy, ultimately yielding superior clinical efficacy outcomes in the future.

## 1. Introduction

HBV is a small, partly double-stranded DNA virus belonging to the *Hepadnaviridae* family and the *Orthohepadnavirus* genus [1,2]. It causes both acute and chronic hepatitis in humans [2,3,4]. Although massive vaccination efforts have demonstrated efficacy in significantly decreasing the occurrence of new HBV infections, roughly 1.5 million individuals still acquire new infections annually [5]. The diagnosis of HBV infection entails the assessment of serological markers, specifically the levels of viral surface antigen (HBsAg) and the detection of immunoglobulin M antibodies targeting the viral core antigen (IgM anti-HBc) [6,7,8]. The treatment landscape for CHB infections has evolved significantly. The introduction of interferon alpha-2b (IFN-*α*) in 1991 represented a significant milestone in HBV management, and the licensing of peginterferon alpha-2a (pgIFN-*α*) in 2005 further refined interferon therapy. The HBV genome replicates via reverse transcription (RT) of its pregenomic RNA and nucleos(t)ides (NUCs) are inhibitors of HBV RT [3,9,10]. Upon the discovery of NUC analogs, Lamivudine’s approval in 1998 marked the beginning of a new era in HBV treatment. Subsequently, five NUCs were approved: adefovir in 2002, entecavir in 2005, telbivudine and clevudine in 2006, and tenofovir in 2008. These additions have provided healthcare providers and patients with more alternatives for the treatment of CHB infection. The safety and tolerability characteristics of IFN-*α* analogs impose limitations on the duration of treatment, which seldom leads to the elimination of HBsAg or conversion to anti-HBs antibodies [11,12,13]. However, NUCs require long-term administration, which may extend throughout the lifetime of an individual. This extended usage poses a heightened risk of viral resistance upon termination as well as the potential for recurrence of viral replication. The primary reason for the failure of NUCs to produce a functional cure is that they do not target the viral cccDNA present in infected hepatocytes [13], indicating the need for novel antiviral strategies to focus on the pivotal stages of viral life, with the objective of achieving a functional cure that leads to desirable clinical results, including the reduction of HBsAg, seroconversion to anti-HBs, and eradication of cccDNA without the need for long-term drug usage [10].

Novel non-NUC antiviral agents that target various stages of the HBV life cycle have been developed to achieve a functional cure for CHB infection [14,15,16,17,18]. Extensive research and development activities have focused on targeting nucleocapsid assembly, with a notable increase in publications and patent filings in this area. Encapsidation is a crucial phase in the HBV life cycle, since it involves encapsulation of the viral pre-genomic RNA-DNA polymerase complex with 120 core protein (Cp) dimers to form a protective nucleocapsid. The nucleocapsid plays a crucial role in the delivery of the pgRNA template required for reverse transcription, which ultimately results in viral DNA synthesis. Disruption of capsid assembly by CAMs can inhibit viral particle production and prevent the formation of new cccDNA [19,20,21]. The cccDNA reservoir is pivotal for the persistence of CHB infection and poses a challenge to existing therapeutic strategies. Targeting capsid assembly through CAMs has emerged as an innovative approach since this disruption can contribute to the perturbation of the cccDNA pool [22].

**Figure 1:**
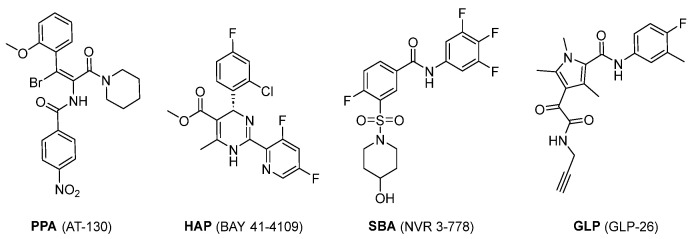
Representative examples from each class of CAMs.

The development of CAMs has progressed through key stages: the discovery of phenylpropenamides (PPAs) in 1998, the introduction of heteroaryldihydropyrimidines (HAPs) in 2002, followed by the sulfamoylbenzamides (SBAs) in 2013, and the recent introduction of glyoxamoylproloxamides (GLPs) in 2020 (Figure 1) [23,24,25,26,27]. Among the discovered CAMs, SBA CAMs have been extensively studied. This can be attributed to the straightforward structural design of compounds like NVR 3-778 and AB-423, supported by compelling preclinical results. Additionally, the accessibility of the co-crystal structures of SBAs with capsids further enriches this avenue of study [10,28,29].

This review article provides a concise explanation of the role of the HBV Cp, its impact on viral replication, and the principles related to the disruption of capsid assembly by CAMs. We also summarize the ongoing refinements in sulfamoyl-based CAMs, highlighting their improved effectiveness against HBV infections through a thorough analysis of patents and the literature. The sulfamoyl-based CAMs featured in this study were categorized into six distinct groups based on their core structural characteristics: SBAs, sulfamoyl bicyclic carboxamides, sulfamoyl aliphatic heterocyclic carboxamides, sulfamoyl aromatic heterocyclic carboxamides, cyclic sulfonamides, and non-carboxamide sulfamoyl-based CAMs. The inclusion of illustrative examples for each of these specified categories will further deepen the understanding of the structural variations and functional attributes inherent in these compounds, highlighting their potential significance as antiviral therapeutics against HBV.

## 2. HBV Life Cycle

**Figure 2:**
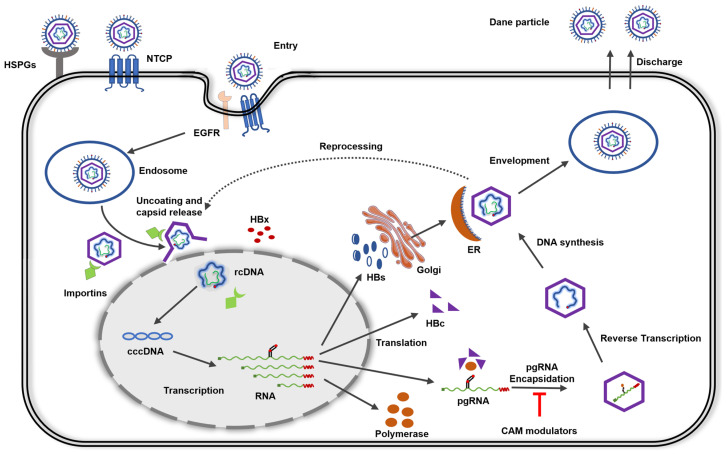
This diagram depicts the basic steps in the life cycle of the human hepatitis B virus. Sodium taurocholate co-transporting polypeptide (NTCP); relaxed circular DNA (rcDNA); covalently closed circular DNA (cccDNA); pre-genomic RNA (pgRNA); endoplasmic reticulum (ER); heparan sulfate proteoglycans (HSPGs); HBV core protein (HBc); epidermal growth factor receptor (EGFR).

HBV infection begins with the attachment of the virus to the host cell surface through low-affinity interactions with heparan sulfate proteoglycans (HSPGs) such as glypican 5 (Figure 2). These interactions are followed by a more precise and high-affinity interaction with the host receptor sodium taurocholate co-transporting peptide (NTCP/SLC10A1). NTCP is a transmembrane transporter that is primarily expressed on the basolateral membrane [30,31,32,33,34]. The internalization of HBV virions occurs through clathrin- or caveolin-mediated endocytosis pathways facilitated by the cofactor receptor tyrosine kinase and epidermal growth factor (EGFR), which directly interact with NTCP [35,36]. Upon release into the cytoplasm, the nucleocapsid, which contains the viral genome, interacts with importin-*β*, facilitating its transportation to the nuclear pore complex. The nucleocapsid undergoes disassembly at this specific location, resulting in the release of the viral genome, referred to as rcDNA, within the nucleus [37,38,39,40].

Inside the nucleus, the host repair system converts rcDNA into stable cccDNA that resembles a minichromosome and is bound by nucleosomes. The function of cccDNA is to produce four distinct viral RNA species through transcription. The lengths of these viral RNA molecules are 3.5 kilobases (kb), 2.4 kb, 2.1 kb, and 0.7 kb [41,42,43]. The transcriptional regulation of these viral RNA species appears to be coordinated by a number of epigenetic factors, including those that modulate histone acetylation and methylation, as well as HBx and Cp [44]. The 3.5 kb pgRNA undergoes translation to produce the capsid protein and polymerase. In addition, pgRNA serves as a template for reverse transcription, leading to the formation of HBV DNA [45,46]. The initiation of encapsidation, which involves the packaging of pgRNA and polymerase into the viral capsid, is triggered by a cis-acting region on pgRNA referred to as epsilon (ε), which matures through complex process within the confines of the nucleocapsid [47,48,49,50,51,52]. This dual function plays a key role in the spread of the virus. Following encapsidation, the polymerase commences the process of reverse transcription, wherein the template known as pgRNA undergoes degradation facilitated by the activity of RNase H, prior to the initiation of plus strand synthesis [21,53,54,55] Circular genomic DNA is generated through multiple rounds of strand transfer. Virion maturation occurs in the endoplasmic reticulum–Golgi complex, where genome-containing nucleocapsids interact with envelope proteins to create mature virions. Mature infectious virions, referred to as Dane particles, exit infected cells via the multivesicular body pathway [53,54,55,56]. Interestingly, the fully developed capsid employs an alternate route to re-enter the nucleus, contributing to rcDNA recycling and replenishing the cccDNA pool [53,54,57].

## 3. Structure and Function of the HBV Cp

**Figure 3:**
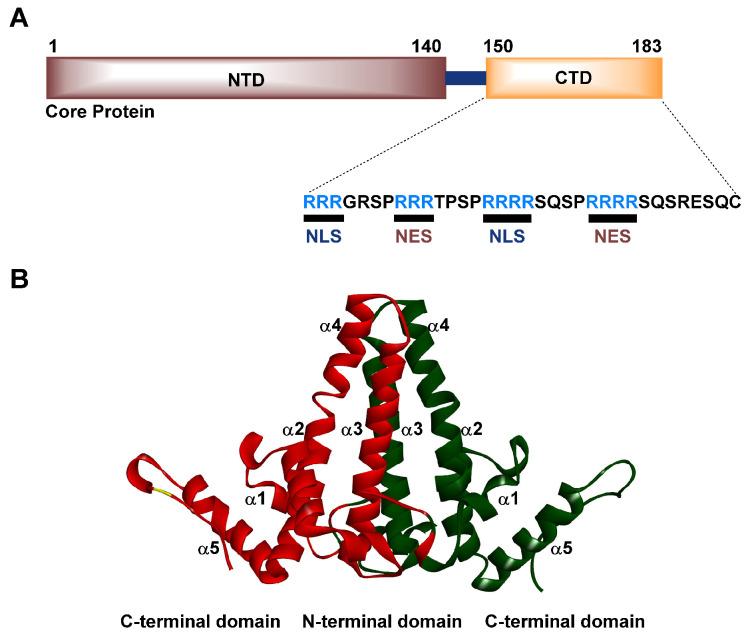
Schematic representation of the monomer and dimer forms of the Cp. (**A**) The Cp monomer with three distinct domains (a detailed explanation is given in the description). (**B**) The Cp dimer structure was obtained from the Protein Data Bank (PDB code 1QGT).

The multifunctional Cp consists of two domains divided by a nine amino acid flexible linker: the N-terminal domain (NTD) and the C-terminal domain (CTD) (Figure 3A). The NTD is responsible for constructing the protective capsid shell, while the CTD is rich in arginine (16 residues) and features seven phospho-acceptors (six serine and one threonine residue), which assumes a crucial function in encapsulating viral genetic material and facilitating DNA maturation throughout the process of virus replication [21,50,51,58]. Interestingly, the Cp can move between the cytoplasm and nucleus because of the nuclear export signals and nuclear localization signals in its CTD. Recent studies have highlighted the significance of the CTD and linker region in viral genome replication and capsid assembly [59]. The HBV capsid is composed of 90–120 Cp dimers that undergo assembly (T = 4 icosahedral symmetry) to form a structure that shields the viral genome during transport to the nucleus. This assembly process is facilitated by weak hydrophobic interactions, with binding energies estimated to be −2.9 kcal/mol and −4.4 kcal/mol. Interfering with these interactions through CAMs has emerged as a promising strategy for the development of anti-HBV drugs [21,50,51,58]. The NTD and linker Cp residue, consisting of amino acids 1–149, demonstrate spontaneous capsid formation in bacterial systems, specifically in *Escherichia coli*. However, their behavior in mammalian cells and cell-free systems, particularly those utilizing rabbit reticulocyte lysate systems, fails to exhibit the process of capsid construction. These findings indicate that the ability of Cp149 to produce capsids is limited to a bacterial system [60,61,62]. The crystal structure of Cp contains several alpha helical regions, with each monomer characterized by five alpha helices [52]. Among these, *α*1, *α*2, and *α*5 are primarily involved in forming a hydrophobic core (Figure 3B). As the monomers dimerize, their amphipathic *α*3 and *α*4 helices combine to form a four-helix bundle that emerges as a distinctive spike shape from the capsid’s surface.

## 4. Influence of CAMs on Capsid Assembly: Classification of CAMs

**Figure 4:**
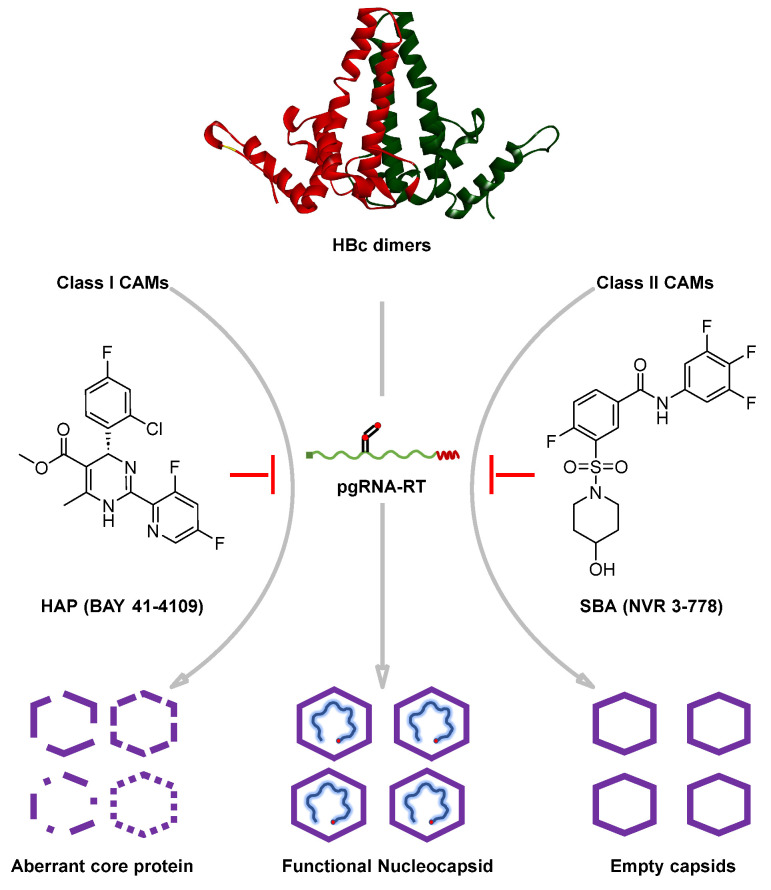
Schematic diagram of the mechanism of CAMs.

In the late 1990s, different chemotypes of drugs were developed to inhibit replication by reducing the capsid levels. These chemotypes included HAPs, PPAs, and SBA derivatives. CAMs can be classified into two distinct phenotypes based on their structural properties and the disruption mechanisms observed in vitro and in hepatocytes [29,63,64]. Wang et al. provided compelling evidence that CAMs effectively impede the formation of viral particles containing both HBV DNA and RNA. In contrast, NUCs have a more targeted mechanism of action, specifically disrupting the synthesis of viral particles containing HBV DNA. This study underscores the differential mechanisms of action of these compound classes, offering valuable insights into their specific roles in modulating various facets of the HBV replication process [65]. The two primary categories of CAMs are class I HAPs and class II PPAs and SBA derivatives, which have distinctive effects: HAPs induce abnormal capsid formation, whereas SBAs generate capsids with typical morphology but devoid of pgRNA (Figure 4) [63].

Both categories of CAMs possess the same target, specifically the hydrophobic pocket positioned at the dimer–dimer interface. Their mode of action centers on disrupting Cp assembly, thereby impeding the generation of infectious virions [29,63,64]. This disruption involves alteration of the conformational state of the Cp, which is achieved through thermodynamic adjustments involving modifications in pairwise contact energies or kinetic adjustments involving changes in nucleation rates. These alterations promote the formation of large, aberrant polymers that lack encapsulation, rendering them highly vulnerable to degradation and intracellular aggregation [66,67]. Class I CAMs such as HAPs enhance Cp assembly and induce malfunctions, leading to the formation of unusual structures [68,69,70], whereas Class II CAMs like SBAs, accelerate connections between Cps, producing empty capsids that lack genetic material. Understanding the impact of energy and speed on virus assembly is essential for comprehending the functioning and potential resistance of viruses. The stability of the connections between Cp units directly influences the effectiveness of CAMs in halting the assembly process [67,71,72,73,74]. Furthermore, certain CAMs not only restrict the synthesis of rcDNA but also impede the creation of *de novo* cccDNA by interfering with the uncoating of nucleocapsids during the first stages of the viral life cycle [75].

### 4.1. Frequently Used In Vitro Cell Culture Models for HBV Infection Studies

The development of CHB cell culture models over the past two decades has significantly contributed to our understanding of HBV replication dynamics and the development of anti-HBV drugs [76]. Frequently used in vitro cell culture models for HBV infection are highlighted in Table 1. At present, two main cell culture models are commonly employed for in vitro research on HBV: primary human hepatocytes (PHHs) and cell lines derived from hepatomas [77,78,79,80]. However, existing models encounter difficulties in effectively maintaining the complete viral life cycle of HBV during in vitro virus spread [76].

PHHs can be carefully grown from aborted fetal liver embryos or from the periphery of tumors in patients who have had their liver resections [77,81]. These hepatocytes facilitate the entire life cycle of HBV infections and perform normal liver functions, such as polarizing hepatocytes and producing bile [78]. PHHs have several hepatocyte-specific host factors and a fully functional innate immune system that combats infections [82].

In the context of HBV infection research, PHHs emerge as a preeminent in vitro culture system due to their unparalleled physiological fidelity [80,82]. However, their widespread use is limited due to low availability, rapid loss of differentiation status, susceptibility to HBV infection shortly after plating, and significant variability from donor to donor [83]. The processes governing the differentiation and dedifferentiation of PHHs are still not well understood [84].

Several hepatoblastoma-derived cell lines, including HepaRG [80], HuH7 [84], and HepG2 [79], have been extensively utilized for in vitro investigations of HBV infections. HepaRG cells, originating from a liver tumor infected with chronic Hepatitis C, exhibit similarities to PHHs in terms of hepatocyte polarization, bile formation, NTCP expression, and fully functional innate immune responses [79,80,84]. Despite these parallels, HepaRG cells receive less research attention due to their reduced HBV infection rate and limited cell-to-cell transfer capabilities [32,85,86,87]. HuH7 and HepG2 cells, isolated from hepatocellular carcinoma, are transiently transfected with HBV DNA to initiate the viral replication cycle, facilitating HBV particle secretion [34,84,88]. The utility of a polarized HepG2 cell line for sustained HBV genome expression has been demonstrated. Distinct cell lines derived from HepG2, namely HepAD38 [89], HepG2.2.15 [79], and HepG2.117 [90], serve specific purposes. HepAD38 utilizes a tetracycline-regulated system to control HBV pgRNA expression. HepG2.2.15, a stable and passagable cell line, serves as a reliable source of infectious particles, enabling diverse assays and the evaluation of antiviral drugs. Similarly, HepG2.117 is designed to express the entire HBV genome [79,89,90]. The introduction of NTCP-overexpressing hepatoma cell lines, such as HuH7-NTCP and HepG2-NTCP, following the 2012 discovery of NTCP as an HBV infection receptor, has significantly advanced studies on cell entry inhibitors [34]. Recent advancements in the HepG2-NTCP sec+ cell line have contributed to in-depth understanding of HBV infections, offering a novel perspective in research [91].

### 4.2. Sulfamoyl Benzocarboxamides as CAMs

**Figure 5:**
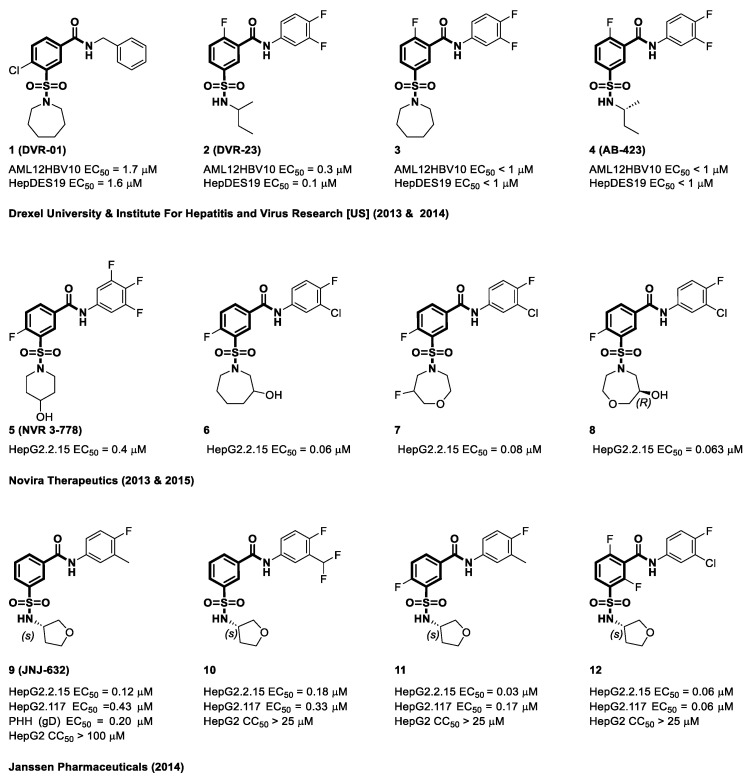
The chemical structures of sulfamoyl benzocarboxamide CAMs.

The introduction of the first SBAs in 2013 by Campagna et al., affiliated with Drexel University and the Institute for Hepatitis and Virus Research [US], represented a significant breakthrough [27]. Campagna et al. conducted primary screening of a library of 26,900 molecules at a concentration of 10 μM, leading to the identification of 120 compounds capable of reducing HBV core DNA levels. In this pool, 40 compounds exhibited remarkable capacity to selectively impede HBV replication, which was evidenced by EC_50_ values below 10 μM while maintaining acceptable cytotoxicity levels at concentrations up to 50 μM. Of the 40 hits, 36 showed a shared structural characteristic, identified as a multi-substituted phenyl structure accompanied by two unique side chains consisting of an amide and a sulfonamide functional group. After this initial success, their focus shifted to exploring SBA derivatives that inhibit HBV replication. An extensive structural similarity search encompassing 87,000 drug-like molecules identified 591 SBA derivatives, which were categorized into two groups distinguished by their substitution patterns at the amide linker: Group 1 (Figure 5, **1**) and Group 2 (Figure 5, **2**). Interestingly, throughout their SAR studies, the central cyclic core linked to an N-substituted phenyl carboxamide remained the same in nearly all the compounds. The primary structural modifications were predominantly observed in the central phenyl rings and side chains containing sulfonamide functional groups. The antiviral effectiveness of DVR-23 was evaluated using HepDES19 and AML12HBV10 cell line assay systems. These findings indicate that the compounds showed minimal cytotoxicity in both cell lines at concentrations > 50 µM. The effective concentrations at which 50% of the cells were affected (EC_50_) were 0.3 µM and 0.1 µM for the two cell lines [27,92]. In June 2013, Lam et al. from Novira Therapeutics made a significant advancement with the introduction of a novel preclinical capsid assembly modulator called NVR 3-778 [29]. In in vitro tests, NVR 3-778 demonstrated strong antiviral activity by effectively suppressing the replication of HBV in HepG2.2.15 cells (EC_50_ = 0.4 µM) [29,93].

Since 2014, dynamic research on SBAs has yielded invaluable insights into the SARs of diverse components linked to sulfonamide side chains and changes in core substituents [94,95,96,97,98,99,100,101,102,103,104,105,106,107,108]. In 2014, Janssen researched sulfamoyl aryl carboxamides, focusing on the aniline portions of amides and the amine components of sulfonamides. This effort led to a lead compound featuring tetrahydrofuran (**9**–**12**), which was published in 2018. Their research involved a comprehensive SAR analysis using five SBA derivatives carefully selected from their own compound library. Employing a systematic hit-to-lead optimization approach, the researchers effectively synthesized JNJ-632 (**9**), a remarkably strong inhibitor of HBV DNA replication. This compound exhibited an EC_50_ value 0.12 µM and a CC_50_ value surpassing 50 µM in HepG2.2.15 cells, and EC_50_ = 0.43 µM in HepG2.117 cells [94,95]. In 2014, the Institute for Hepatitis and Virus Research [US] collaborated with Drexel University and Enantigen to develop a patent application centered on SBAs. Some of these structures are illustrated in Figure 5 (**3** and **4**). The antiviral efficacy of these compounds was measured using the AML12HBV10 and HepDES19 cell lines. Notably, both compounds **3** and **4** demonstrated EC_50_ values less than 1 µM, indicating their potent antiviral activity in the AML12HBV10 and HepDES19 cell lines [96].

**Figure 6:**
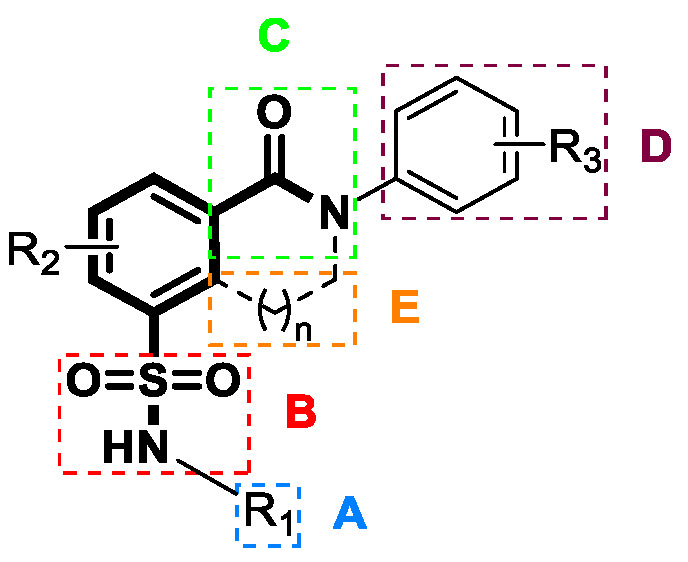
Markush Structure (representative chemical structure) of SBAs by Ozkan Sari and colleagues at Emory University.

**Figure 7:**
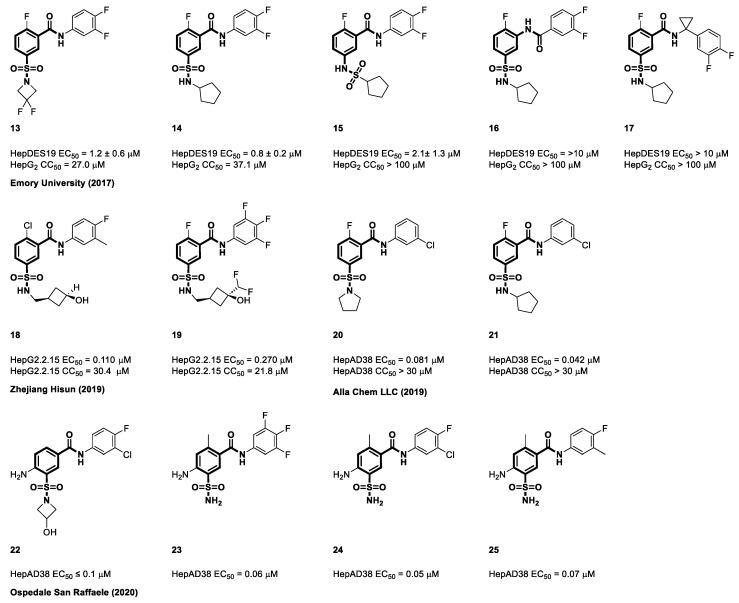
The chemical structures of sulfamoyl benzocarboxamide CAMs.

Subsequently, Novira expanded their research efforts to investigate sulfonamides, with a particular focus on examining sulfonamide derivatives that incorporate substituted azepanes, as well as related analogs such as oxazepanes and diazepanes. The antiviral efficacy of these compounds against HBV replication was evaluated in a hepatoma cell line that was genetically modified to produce HBV. Significantly, within this specific series, three compounds (**6**–**8**) demonstrated noteworthy antiviral efficacy, with EC_50_ values of 0.06 µM, 0.08 µM, and 0.063 µM [97]. This study, authored by Kuduk et al., was published in 2019. In addition, the racemate molecule (**8**) exhibited improved anti-HBV activity as well as reduced cytotoxicity in HepG2.2.15 cells (19 µM). This study represents a significant step towards the pursuit of more effective and safer antiviral compounds [98]. Sari et al., at Emory University, also simplified the SAR analysis of SBAs by dividing the chemotypes into five separate segments, as illustrated in Figure 6 [99]. Compounds **13**–**17** in Figure 7 show how scientists changed the functional groups and substituents inside these chemical structures. Derivative **13** showed strong antiviral activity at notably low micromolar doses, as indicated by an EC_50_ value of 1.2 µM in HepDES19 cells. The replacement of difluoro azetidine showed significant prominence. The antiviral activity of compound **15** was diminished by inversion of the sulfonamide group. However, modifications to sections C and D, specifically compounds **16** and **17**, resulted in a significant decrease in antiviral efficacy [99].

In 2019, Cao et al. introduced a series of compounds distinguished by the presence of cyclobutanol as a solvent-exposed end group within the sulfonamide side chains [100]. Compounds **18** and **19** of the present invention exhibited significant antiviral activity, with EC_50_ values ranging from 0.11 to 0.27 µM. Additionally, these compounds exhibited comparatively low cytotoxicity, with CC_50_ values ranging from 30.4 to 21.8 µM, when tested on HepG2.2.15 cells. Compound **19** showed favorable pharmacokinetic and toxicity results, making it more likely to be an effective and safe drug (T_1/2_ = 16 ± 3 h, AUC_0–t_ = 8397 ± 461 h.ng/mL, AUC_0–inf_ = 13467 ± 1030 h.ng/mL, CL = 2.6 ± 0.2 mL/Kg/min and Vss = 3.59 ± 0.12 L/Kg) [100].

In 2019, Ivachtchenko et al. achieved major advancements in the field of capsid inhibitors, which have the potential to be used as chemotherapeutic drugs to treat HBV infections [101]. The inhibitors represented by **20** and **21** were found to show significantly high levels of activity, as evidenced by their EC_50_ values of 0.081 μM and 0.042 μM, respectively. The results of their study showed that the efficacy of the tested chemicals surpassed that of the strongest known HBV inhibitors [101]. A research group at Ospedale San Raffaele explored the incorporation of substituents into the central core structure. The examination specifically targeted locations neighboring the two crucial pharmacophoric side chains. The approach employed by the researchers entailed the introduction of an amine functional group next to the sulfonamide side chain, parallel to the carboxamide side chain. Notably, Novira’s compound NVR 3-778 exhibited a fluorine substitution in a comparable location. Remarkably, this investigation led to the discovery of four compounds in this series, designated **22**, **23**, **24**, and **25**, that all displayed an impressive anti-HBV action, with EC_50_ values starting at 0.1 μM in HepAD38 cells [102]. In China, Lv et al. introduced structural changes in the 4-hydroxypiperidine moiety, resulting in the synthesis of three unique series of SBA derivatives [103]. Among the series under investigation, compound **26** exhibited remarkable potency, as evidenced by its IC_50_ value of 0.21 µM in the HepAD38 cell line. Surprisingly, compound **26** showed lower cytotoxicity (7.41 µM) than NVR 3-778 while still maintaining its anti-HBV efficacy. Further replacement within the piperidine ring while retaining the hydroxyl group led to the synthesis of **28**, which demonstrated an EC_50_ value of 0.3 µM. Its derivative, **27**, exhibited an EC_50_ value of 0.39 µM. Significantly, compound **29** exhibited noteworthy pharmacokinetic characteristics in mice when administered at a dosage of 50 mg/kg (T_1/2_ = 7.79 ± 0.77 h, T_max_ = 0.83 ± 0.28 h and AUC_0–t_ = 232025 ± 46728 h.ng/mL) [103]. NVR 3-778 progress could be hampered by its restricted water solubility of 0.003 mg/mL at a pH of 2. To address this constraint, Kai LV and colleagues developed an innovative category of amino acid prodrugs for NVR 3-778. Compound **31**, an ester prodrug of NVR3-778 derived from L-valine, showed a notable enhancement in aqueous solubility, reaching a concentration of 0.7 mg/mL at a pH of 2. In addition, researchers have successfully synthesized alanine and phenylalanine derivatives (**30** and **33**) and observed favorable tolerance. All derivatives maintained a similar level of IC_50_ value of 0.28 to 0.56 µM in the HepG2.2.15 cell line, with CC_50_ (>10 µM) [104].

**Figure 8:**
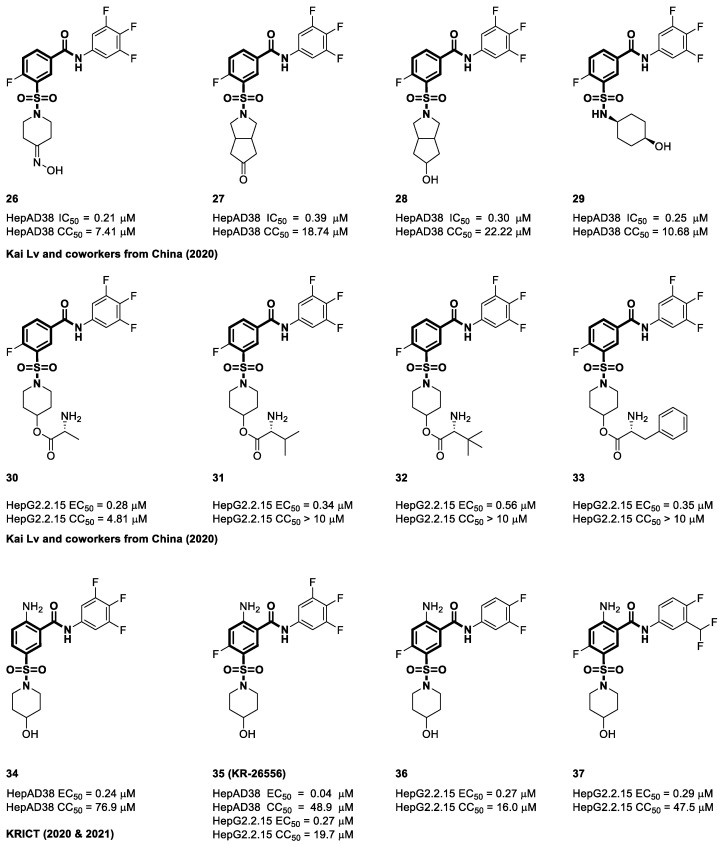
The chemical structures of sulfamoyl benzocarboxamide CAMs.

A study conducted in 2020 by Na et al. from the Korea Research Institute of Chemical Technology (KRICT) aimed to develop SBAs with the objective of hindering viral replication by focusing on inhibiting capsid formation prior to polymerase encapsulation [105]. The research team initiated the development of novel HBV CAMs, inspired by the structural characteristics of NVR 3-778. By employing a strategic methodology that integrated molecular hybridization and bio-isosteric principles, the researchers successfully optimized the structure of NVR 3-778, resulting in the development of a highly effective compound known as KR-26556 (**35**) shown in Figure 8. This heightened potency was demonstrated by an impressive EC_50_ value of 0.04 µM in HepAD38 cells, which was roughly nine times greater than that of NVR 3-778. Importantly, **35** showed enhanced efficacy while maintaining a CC_50_ of 48.9 µM [105]. The efficacy of **35** in reducing HBV DNA levels was evident in HepAD cells, highlighting its potential as an antiviral agent. However, its efficacy was significantly lower in HepG2.2.15 cells, with an EC_50_ of 0.27 μM and a CC_50_ of 19.7 μM, underscoring the critical need for novel anti-HBV inhibitors. A subsequent follow-up study led by Lee et al. at KRICT aimed to expand the capabilities of **35** by synthesizing derivatives and exploring their potential as CAMs [106]. This study aimed to establish the connection between structural variations and antiviral activity and yielded compounds **36** and **37**, revealing that the presence of three fluorine atoms, a shared feature of compounds **35** and NVR 3-778, is not a strict prerequisite for antiviral effectiveness. Notably, transmission electron microscopy analysis of **36** revealed that it could induce the formation of atypical HBc particles. Over a 12 h period, these particles transformed from spherical to rod-like shapes and eventually evolved into tubular and rod-shaped structures within 3–5 days, signifying their pivotal role in disrupting HBV assembly. These findings provide a promising starting point for future studies to promote the development of safer and more potent CAMs for treating HBV infection [106].

**Figure 9:**
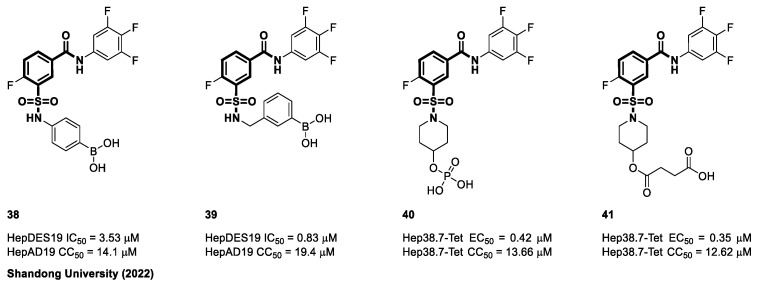
The chemical structures of sulfamoyl benzocarboxamide CAMs.

In 2022, Wang et al. from Shandong University in China reported the modification of NVR-3778 compounds [107]. The solvent-exposed region of these derivatives was modified to include phenylboronic acid (**38**) or phenylboronate ester groups as mentioned in Figure 9. The introduction of a methylene group led to the formation of compound **39**, with an EC_50_ value of 0.83 μM and a CC_50_ value of 19.4 μM. Moreover, compound **39** demonstrated an improved level of solubility in aqueous solution, as evidenced by a measured value of 328.8 μg/mL at pH 7 (solubility of NVR 3-778, 35.8 μg/mL at pH 7). Pharmacokinetic assessments performed in Sprague Dawley rats yielded positive outcomes for the terminal half-life (T_1/2_ = 1.08 h) and absorption rates (T_max_ = 0.38 h). However, the clearance rate of the orally administered drug was significantly higher than that of the intravenously administered drug. As a result, bioavailability was only 2.85% [107]. Further efforts by the same laboratory to make NVR 3-778 more soluble in water led Xiangkai et al. to synthesize many unique prodrugs of phosphate (**40**) and carboxylic acid (**41**). Based on in vitro HBV replication studies, the prodrugs’ antiviral efficacy (EC_50_ = 0.35–0.42 μM) was not compromised. In three phosphate buffers with different pH values (2.0, 7.0, and 7.4), prodrug **41** was hundreds of times more water-soluble than NVR 3-778. Additionally, **41** showed good pharmacokinetic property in rats (T_1/2_ = 1.92 ± 0.227 h, C_max_ = 747 ± 104 ng/mL) [108].

### 4.3. Sulfamoyl Bicyclic Carboxamide-Based CAMs

Identification of more chemotypes of CAMs may yield additional prospects for novel CHB therapies. These novel chemotypes may alter the kinetics or thermodynamics of capsid assembly in different ways, yielding varying benefits against HBV in comparison with the currently available CAMs [109]. Pyrrolidine-derived bicyclic cores were first discovered by Janssen. These cores consist of saturated rings in which the nitrogen atom is connected to either the sulfonyl or carbonyl groups. They are analogs of beta-proline and exhibit bicyclic structures with two interconnected side chains. The central core phenyl ring of NVR 3-778 was substituted with 1λ^2^-indoline (**42**), 2λ^2^-azabicyclo [3.1.0] hexane (**43**), 3λ^2^-azabicyclo [3.1.0] hexane (**44**), and (1s,4s)-7 λ^2^-azabicyclo [2.2.1] heptane (**45**) to evaluate the antiviral efficacy. Stably transfected cell lines, specifically HepG2.2.15 and HepG2.117, were selected as the preferred models for antiviral testing because of their consistent and substantial secretion of HBV virion particles associated with both acute and chronic infections. In the HepG2.2.15 cell line, the examined compounds displayed EC_50_ values of 2.0 µM, 2.8 µM, 2.39 µM, 0.61 µM, and 0.58 µM, respectively. Similarly, in the HepG2.117 cell line, the EC_50_ values were 3.9 µM, 2.0 µM, 2.65 µM, 4.7 µM, and 0.85 µM for the same compounds. Notably, these compounds exhibited low cytotoxicity, with CC_50_ values exceeding 25 µM in HepG2 cells [110].

**Figure 10:**
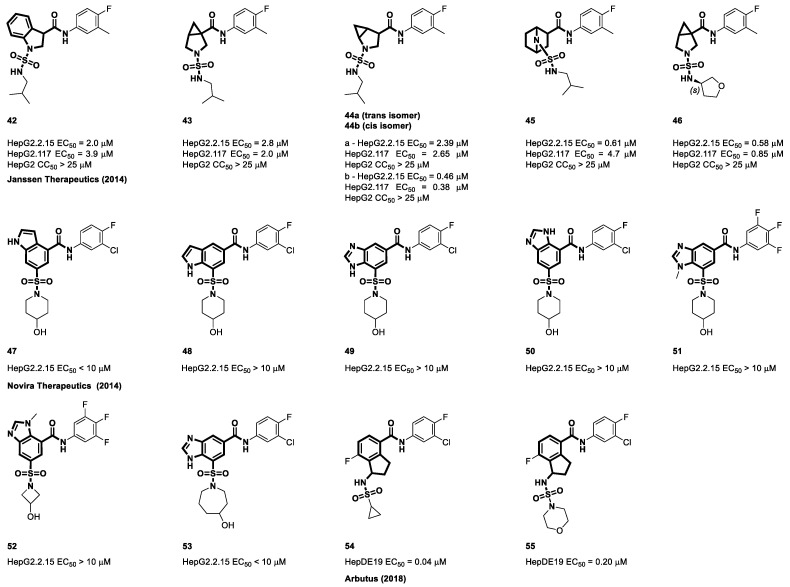
The chemical structures of sulfamoyl bicyclic-carboxamide CAMs.

Novira’s research continued by investigating the inclusion of an extra ring fused to the primary phenyl core. This resulted in the formation of molecules containing fused heterocyclic rings, such as indoles, benzo[d]imidazoles, and *N*-methyl-benzo[d]imidazoles. Novira’s researchers tested the chemotypes at four different concentrations, specifically 10 µM, 3 µM, 1 µM, and 0.3 µM, to determine their impact on the process of capsid formation. Compounds **48** to **50** in this collection of chemicals demonstrated notable efficacy in the assembly test. They showed EC_50_ values less than 10 µM in the HepG2.2.15 cell line, indicating that they were primary hits. Many positional combinations of the heterocyclic ring, particularly in connection with the phenyl ring, have also been investigated in subsequent studies. This was observed for compounds **47** to **53**, as shown in Figure 10 [111]. In 2018, the Arbutus group studied the utilization of a fused ring 2,3-dihydro-1H-indene as an alternative to the central phenyl ring. Using a HepDE19 assay, the inhibitory effects of the selected compounds were evaluated for their ability to block rcDNA production. The most promising compounds showed EC_50_ values of 0.04 µM (**54**) and 0.2 µM (**55**) [112].

**Figure 11:**
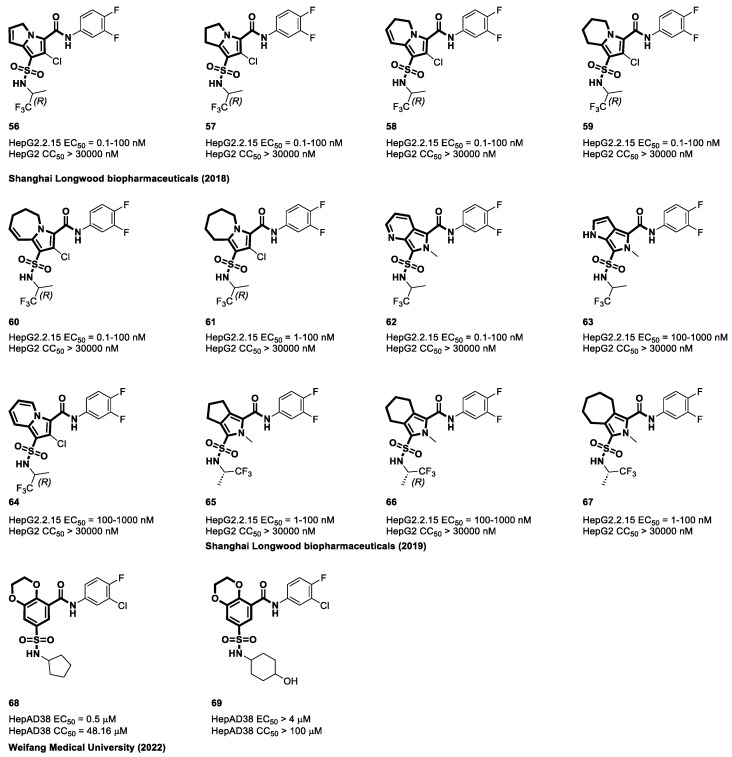
The chemical structures of sulfamoyl bicyclic-carboxamide CAMs.

In 2018, Wang et al. from Shanghai Longwood biopharmaceuticals extensively investigated the potential of bicyclic nucleocapsid inhibitors for the treatment of hepatitis B [113]. These inhibitors featured a diverse range of bicyclic cores, including derivatives such as pyrrolizine (**56**), dihydropyrrolizine (**57**), dihydroindolizine (**58**), tetrahydroindolizine (**59**), dihydropyrroloazepine (**60**), tetrahydropyrroloazepine (**61**), methylpyrrolopyridine (**62**), methyldihydropyrrolopyrrole (**63**), indolizine (**64**), methyltetrahydrocyclopentapyrrole (**65**), methyltetrahydroisoindole (**66**), and methylhexahydrocycloheptapyrrole (**67**). The anti-HBV activity of these different compounds, **56** to **67**, shown in Figure 11, was extensively examined in HepG2.2.15 cells. The strong anti-HBV activity of the compounds was shown by the results of the in vitro experiment; the EC_50_ values ranged from 0.1 to 1000 nM for compounds **56** to **67**. Significantly, the CC_50_ values exceeded 30,000 nM, indicating low cytotoxicity and their potential as promising therapeutic alternatives to hepatitis B [113,114]. The primary aim of the study by Liu et al. at the Weifang Medical University in China was to develop a novel potent capsid protein inhibitor with minimal toxicity and favorable metabolic stability [115]. The study team employed proven principles in drug design, including pharmacophore hybridization, bioisosterism, and scaffold hopping, to design, synthesize, and evaluate dihydrobenzodioxine-based capsid protein inhibitors. Compound **68** exhibits considerable potential as a lead compound, which is primarily attributable to its notable EC_50_ value of 0.50 µM in the inhibition of HBV DNA replication. The compound’s CC_50_ value of 48.16 µM serves as an illustration of its potential cytotoxicity profile. The most notable discovery of this study was the enhanced efficacy of the inhibitor through the incorporation of hydrophobic and nonpolar moieties into the sulfonamide group. Optimal outcomes were achieved when the dimensions of these additional constituents aligned with those of the pentagonal ring [115].

### 4.4. Sulfamoyl Aliphatic Heterocyclic-Carboxamide-Based CAMs

Sulfamoyl aliphatic heterocyclic carboxamides have emerged as important structural components in the development of novel CAMs. These compounds are distinguished by the replacement of an aliphatic heterocyclic ring with a core phenyl ring, making them a promising class of drugs for the regulation of capsid assembly to treat HBV. The discovery of pyrrolidine-based cores with these properties can be credited to Janssen Pharmaceuticals in 2014. Compound **70** from their research demonstrates significant anti-HBV efficacy with EC_50_ values of 0.94 μM in the HepG2.2.15 cell line and 0.72 μM in the HepG2.117 cell line. Furthermore, these compounds showed a favorable cytotoxicity profile, with a CC_50_ value above 25 μM in HepG2 cells [110].

**Figure 12:**
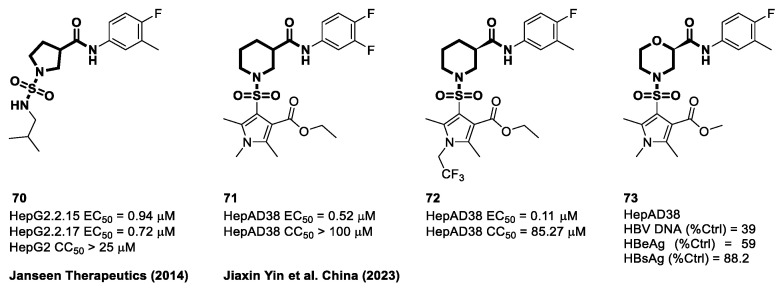
The chemical structures of sulfamoyl aliphatic heterocyclic-carboxamide CAMs.

In a study conducted by Yin et al. at the Chongqing Medical University in China as highlighted in Figure 12, a series of N-sulfonylpiperidine-3-carboxamide (SPC) derivatives were synthesized and evaluated for their anti-HBV potential [116]. Compound **71** demonstrated remarkable efficacy in inhibiting HBV replication in numerous cell lines, including HepAD38, HepG2-HBV1.3, and HepG2-NTCP cells. Compounds **71** and **72** significantly decreased the quantity of HBV DNA in cell culture supernatants to less than 20%. Surprisingly, the antiviral activity decreased when the piperidine moiety (present in **71**) was replaced with morpholine (present in **73**). Compounds **71** and **72** showed activity against HBV DNA with EC_50_ values of 0.52 μM and 0.11 μM, respectively, while exhibiting minimal cytotoxicity with CC_50_ values exceeding 100 μM and 85.27 μM. Electrophoresis experiments revealed that the addition of **71** caused the disintegration of both the HBV capsid and the core protein, a property that neither **72** nor JNJ-632 possessed. Notably, JNJ-632 is a well-known Class II CAM known for its ability to induce the formation of empty, pgRNA-free capsids. On the basis of these findings, researchers hypothesized that compound **72**, similar to JNJ-632, could be classified as Class II because of its effect on HBV capsid and core protein degradation [116].

### 4.5. Sulfamoyl Aromatic Heterocyclic-Carboxamide-Based CAMs

**Figure 13:**
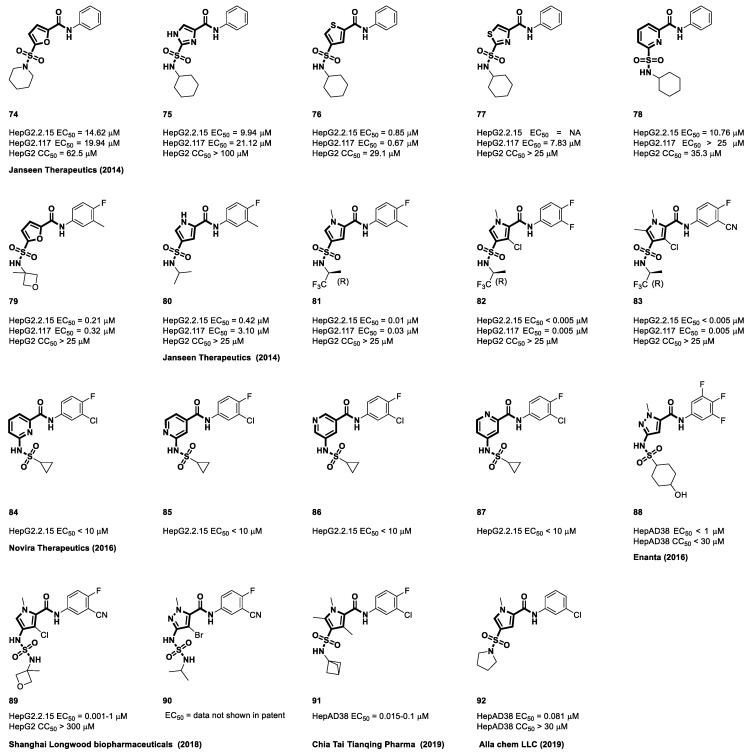
The chemical structures of sulfamoyl aromatic heterocyclic-carboxamide CAMs.

Janssen Pharmaceuticals was the first to report heteroaryl chemotypes such as furan, imidazole, thiophene, thiazole, and pyridine (**74**–**79**). Although they retained important amide and sulfonamide side chains in the same relative positions, the introduction of these various heterocyclic cores resulted in subtle differences in how they bound to their target molecules [94]. Minute deviations in the bond angles across these 5- and 6-membered ring structures provide exciting opportunities to create new compounds with potentially better properties for drug development. The compounds described in this patent exhibited relatively low anti-HBV activity (Figure 13). In their subsequent research efforts, they focused on nitrogen-containing heterocycles, ultimately resulting in the formation and development of a pyrrole-amide series (**80**). The incorporation of pyrrole-derived cores, wherein the amide side chain was positioned alpha to the nitrogen of the pyrrole within this structural scaffold, constituted a notable improvement over prior investigation. In prior studies, compound **74** showed reduced efficacy, as indicated by EC_50_ values of 14.62 μM and 19.94 μM. Significantly, the alkylation of the nitrogen atom in the pyrrole ring and the meticulous choice of amines for the sulfonamide groups (**80**–**83**) seem to have had a crucial impact on enhancing the efficacy of the compounds. Furthermore, the introduction of halogen atoms at the third position of the pyrrole ring, located between the two side chains, plays a significant role in augmenting the effectiveness of these compounds [117].

Novira pursued an innovative approach by altering the configuration of the sulfonamide group and effectively switching the positions of the nitrogen and sulfonyl moiety within the core structure of the molecule. This alteration introduced a novel structural dynamic, particularly by incorporating heteroaryl rings such as pyridine (**84**–**87**). Importantly, they retained the crucial meta-relationship between the important amide and sulfonamide side chains, preserving a key element of the molecular architecture. Additionally, they explored the repositioning of nitrogen atoms in various molecules, considering them as potential substitutes for the phenyl moiety, and evaluated their anti-HBV activities. The compounds detailed in this patent display limited anti-HBV activity [118]. In addition, Enanta Pharmaceuticals’ exploration of NVR 3-778’s phenyl core replacement with pyrazole, along with research into sulfonyl linker substitutions while preserving the meta-connection between side chains, has yielded promising anti-HBV activity results [119]. Compound **88** from the pyrazolamide series, with an EC_50_ value below 1 μM and a CC_50_ value below 30 μM, exhibits remarkable efficacy and low toxicity in HepAD38 cells, presenting a potential therapy for HBV. In 2018, the Shanghai Longwood Biopharmaceuticals patent for a sulphonamide-aryl amide compound to treat HBV involved a sulphonamide-aryl amide core with pyrrole (**89**) and pyrazole (**90**) derivatives. The EC_50_ value for compound **89** reported in their invention as an HBV replication inhibitor ranged from 0.001 to 1 μM, showing that it reduced HBV replication. The compounds in that invention had CC_50_ values more than 30 μM, suggesting that they were extremely non-toxic to normal cells [87]. These compounds outperformed the control chemicals in typical solubility tests and pharmacokinetic studies. The most active representatives of this compound series, **89**, **91**, and **92**, released by Longwood in 2018 and Alla Chem LLC and Chia Tai Tianqing Pharma in 2019, showed substantial improvements over Novira’s work on sulfamoyl aromatic heterocyclic carboxamides [101,114,120].

### 4.6. Cyclic Sulfonamide Carboxamide-Based CAMs

**Figure 14:**
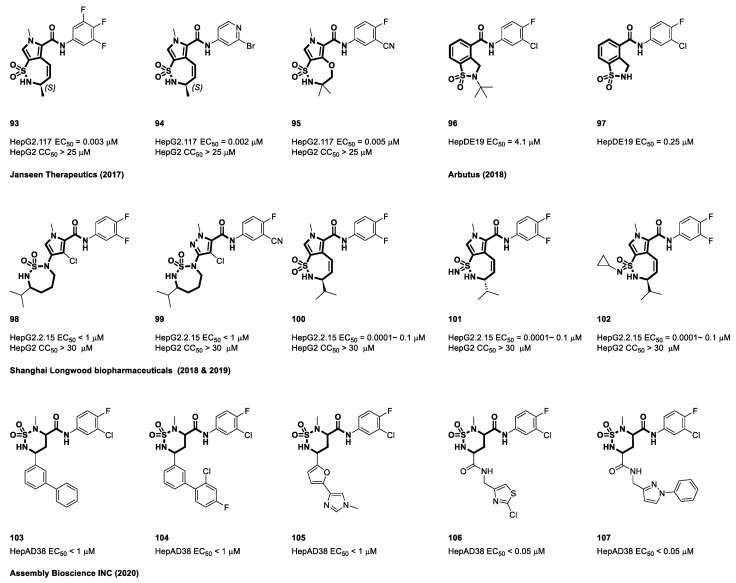
The chemical structures of cyclic sulfamoyl carboxamide CAMs.

Janssen’s initial attempt to incorporate a benzene ring with the five-membered beta-proline did not yield satisfactory results [94]. However, a significant breakthrough was achieved when researchers focused on the pyrrolamide scaffold [117]. They successfully synthesized novel bicyclic cores (**93**–**95**) by cyclizing the sulfonamide side chain to the 3-position of the pyrrole. Notably, cyclic sulfonamide rings have demonstrated the ability to accommodate various substituents without compromising anti-HBV efficacy. The effectiveness of these compounds was evaluated using the HepG2.117 cell line, and cytotoxicity assessments were performed on HepG2 cells. Janssen’s innovative approach underscores the potential of these compounds as candidates in an ongoing effort to combat hepatitis B. Figure 14 provides a visual representation of the most promising compounds identified within the scope of this invention [121].

Furthermore, Arbutus Biopharma has made substantial contributions to this research field through innovative designs. The disclosure provided by the authors encompasses substituted dihydrobenzo[d]isothiazole-4-carboxamide cyclic sulfonamides and their analogs. These compounds exhibited strong inhibitory effects on capsid proteins, significantly impeding the production of rcDNA in HepDE19 cells. Several illustrative examples are provided in Figure 14 (**96**–**97**) [122]. In contrast to Janssen’s endocyclic sulphonamides, Longwood explored a range of other chemical scaffolds with multicyclic cores (**98**–**99**) [123]. Significantly, this company also introduced bioisosteres of JNJ’s bicyclic cores, replacing sulfonamides with sulfonimidamides. The standout compounds from this patent demonstrated remarkable sub-micromolar EC_50_ values ranging from 0.0001 to 0.1 μM (**100**–**102**) [124]. In two patent applications filed in 2020, Assembly Bioscience proposed a novel chemical scaffold known as a cyclic saturated six-membered sulfamide ring. In the HepAD38 cell line, compounds **103** to **105** showed an EC_50_ value of 1 μM. In contrast, compounds **106** and **107** demonstrated significantly increased antiviral effectiveness, with EC_50_ values of 0.05 μM in their second patent. These findings highlight the importance of stereochemistry in the unique actions of both side chains. The assembled compounds had two chiral centers within the central six-membered ring, which significantly affected the angles at which the side chains interacted with the target proteins. This is in contrast to planar benzamides, emphasizing the significance of the distinct structural characteristics of these compounds [125,126].

**Figure 15:**
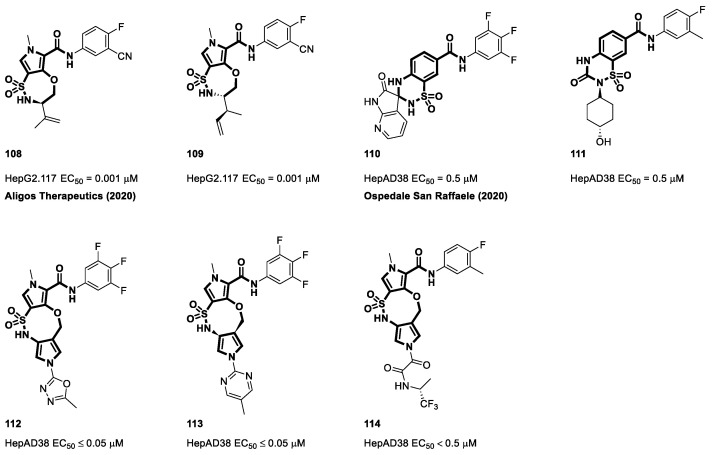
The chemical structures of cyclic sulfamoyl carboxamide CAMs.

In 2020, Aligos Therapeutics reported their findings (Figure 15) on endocyclic sulphonamides derived from pyrrole central cores (**108** and **109**) [127]. Drawing inspiration from Janssen’s work on cyclic sulfonamides with substituents extending from the bicyclic core (**93**–**95**), Ospedale San Raffaele investigated the cyclization of the sulfonamide side chain (**110** and **111**), followed by the cyclization of the sulfonamide side chain into the pyrrole core and added an extra third ring next to the sulfonamide group (Figure 15, **112** and **113**). With EC_50_ values of less than 0.05 μM, compounds **112** and **113** from this patent demonstrated impressive antiviral activity in the HepAD38 cell line. In the same year, Raffaele et al. introduced new compounds that extended a side chain from their tricyclic ring structures using oxamides. The antiviral activity of compound **114**, as indicated in the patent, was observed in the HepAD38 cell line, with an EC_50_ value of less than 0.5 μM [128,129,130].

### 4.7. Non-Carboxamide Sulfomoyl-Based CAMs

**Figure 16:**
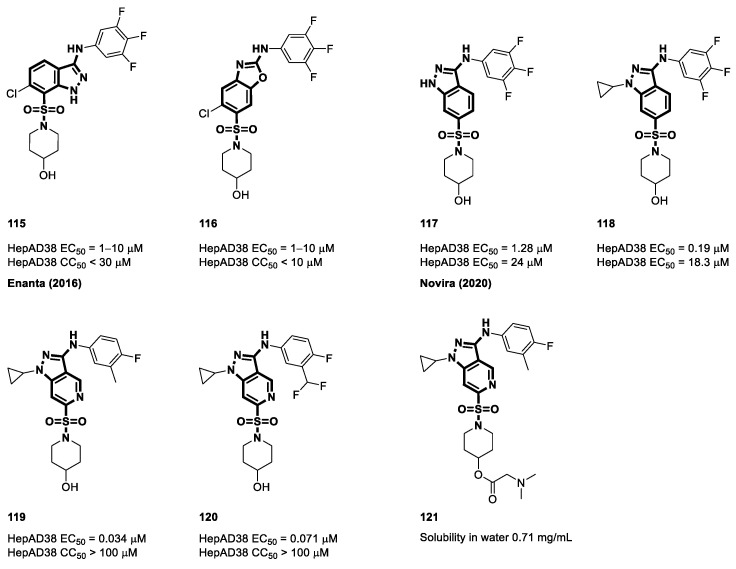
The chemical structures of cyclic sulfamoyl carboxamide CAMs.

In 2016, Qiu et al. and the Enanta Pharmaceuticals research team presented a novel class of compounds characterized by non-carboxamide sulfonamides with distinctive bicyclic structures, specifically indazole and benzo[d]oxazole [119]. Their primary objective was to investigate how the removal of the carbonyl group from carboxamide affects its antiviral properties. Compounds **115** and **116** in Figure 16 showed moderate potency, with EC_50_ values of 1 to 10 μM in HepAD38 cells. Equally significant was the evaluation of compound toxicity, which was conducted by seeding the cells at a density of 15,000 cells per well. The outcome revealed a reassuring result: these compounds demonstrated remarkably low toxicity, underscoring their potential as candidates for the development of effective and safe antiviral agents against HBV [119].

In 2020, Wang et al. applied an innovative conformational constraint approach to the lead compound NVR 3-778 [131]. This approach involved the cyclization of carbonyl and the carbon of the core phenyl ring to create a novel scaffold. Within this innovative scaffold, a nitrogen atom within the aromatic heterocycle was designed to engage in hydrogen-bonding interactions with specific amino acids, replacing the conventional carbonyl interaction. This strategic modification aimed to enhance the binding affinity of the compound and its interaction with its target, potentially leading to improved pharmacological properties and therapeutic efficacy. Indazole and quinazoline scaffold molecules were synthesized to test the validity of these hypotheses. The experimental findings demonstrated that the quinazoline derivatives did not substantially reduce viral replication. Nevertheless, compound **117** emerged as a very effective agent, exhibiting significant inhibition against HBV with a noteworthy EC_50_ value of 1.28 μM in HepAD38 cells. The research team discovered two more compounds (**119** and **120**) that were classified as class II CAMs and showed the ability to enhance the process of capsid assembly using an SAR methodology. The compounds exhibited CC_50_ values beyond 100 μM, indicating their lack of cytotoxicity toward HepG2 cells. Compound **120** and its analogs showed limited oral bioavailability in mouse models because of their rigid and planar molecular structures as well as their elevated sp2 carbon concentration, resulting in a modest F value of 12%. To circumvent this concern, researchers have synthesized prodrugs with amino acids as the foundation to yield soluble compounds containing amino groups. Specifically, the solubility of prodrug **121** in its citric acid salt form increased to 8.13 mg/mL, and its F improved to 87.6% [131].

## 5. Sulfamoyl-Based Carboxamide CAMs in Clinical Development

Currently, three therapeutic modalities, NVR 3-778, JNJ-6379, and ABI-H0731, are under assessment in phase 2 clinical trials [132,133]. AB-423 has entered phase 1 clinical studies. Numerous review articles have extensively scrutinized clinical trials of CAMs in both phases [134,135,136,137,138,139]. To streamline our discussion, we focus primarily on sulfamoyl-based carboxamide CAMs, specifically those for which the chemical structure has been publicly disclosed.

### 5.1. NVR 3-778

**Figure 17:**
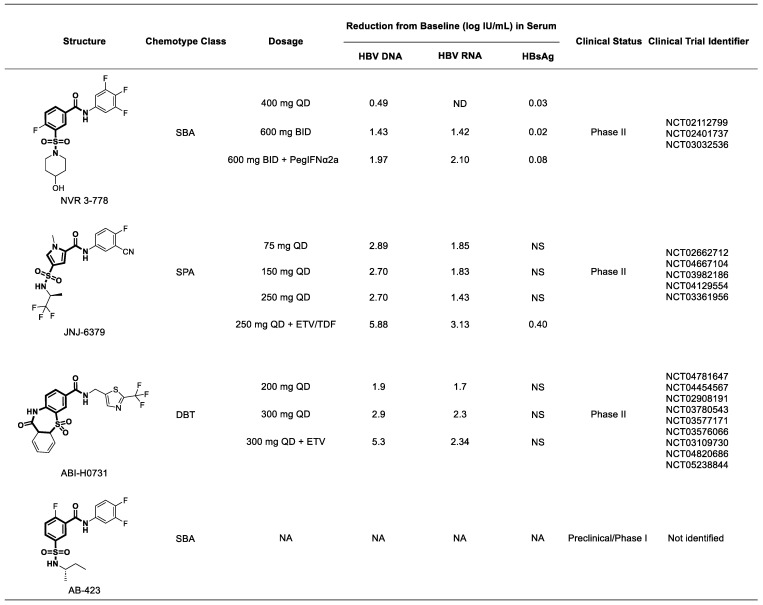
Novel sulfamoyl-based CAMs in clinical trials. BID, twice daily; QD, once daily; HBV, hepatitis B virus; HBsAg, hepatitis B viral surface antigen; NA, not applicable; NS, no significant changes; SBA, sulfamoylbenzamide; DBT, dibenzothiazepinecarboxamide; SPA, sulfamoylpyrolaamide.

NVR 3-778 achieved a notable milestone as a CAM by establishing a clinical proof of concept. In a 28-day trial (Figure 17), it demonstrated promising results in reducing HBV DNA and RNA levels when used in monotherapy and in combination with PegIFN*α*-2a [140]. This SBA CAM, NVR 3-778, is currently advancing through phase 2 clinical trials exploring combinations of various NUCs agents and interferons. In preclinical testing using an HBV-infected humanized uPA/SCID mouse model, the combination of NVR 3-778 with peg-IFNα showed superior inhibition of HBV DNA and RNA in comparison with individual treatments, with no significant improvement in combination with ETV. A substantial reduction in HBV DNA levels was predominantly observed at the high dose of 1200 mg (600 mg twice daily) in these trials. Currently, research is focused on evaluating the combination of NVR 3-778 with peg-IFNα and its NUCs analogs. In a preliminary investigation conducted over a 28-day period, the administration of NVR 3-778 at dosages of 400 and 600 mg, twice daily, as well as 600 mg in combination with peg-IFNα, resulted in notable reductions of 0.49, 1.43, and 1.97 log IU/mL, respectively, in HBV DNA levels. Additionally, the HBV RNA levels decreased by 1.42 log IU/mL in the 600 mg monotherapy group and by 2.10 log IU/mL in the combination therapy group. However, achieving a reduction in HBsAg levels is difficult with CAMs because they do not suppress the transcription or translation processes in the HBV life cycle (NCT02112799 and NCT02401737) [29,141].

### 5.2. JNJ56136379

JNJ56136379, also known as JNJ6379, shows significant efficacy as a type 2 CAM by enhancing core assembly in vitro, resulting in the formation of nucleocapsids devoid of genetic material. The safety and efficacy of JNJ56136379 has been evaluated in a comprehensive two-part phase 1 clinical study (NCT02662712). In part 1 of the study, 30 healthy individuals received either a single oral dose of up to 600 mg or multiple ascending doses of 150 mg for two days, followed by a daily dose of 100 mg once daily for ten days. JNJ56136379 exhibited exceptional tolerability with no dose-limiting toxicities or serious adverse events. Its pharmacokinetics showed dose-proportionality, low clearance, and a notably prolonged half-life ranging from 120 to 148 h, indicating its suitability for once-daily administration. Part 2 of the study involved the oral administration of JNJ56136379 for 28 days at doses of up to 250 mg once daily. During this phase, the drug demonstrated outstanding tolerability and significant antiviral efficacy in treatment-naive individuals diagnosed with CHB. In comparison with the initial measurements, the HBV DNA and RNA levels on day 15 decreased by 1.83 log IU/mL and 1.44 log IU/mL, respectively. Similarly, on day 29, the DNA and RNA levels diminished by 2.70 log IU/mL and 1.43 log IU/mL, respectively [142,143]. The favorable outcomes observed in the initial trials prompted the initiation of phase 2a investigations involving 232 individuals. These trials assessed the efficacy of JNJ-56136379 at doses of 75 and 250 mg when administered in combination with established therapies such as ETV or TDF. In these studies, the control group received monotherapy with NUCs analogs (NCT03361956). Among patients who tested positive for HBsAg, the co-administration of JNJ-56136379 with NUCs resulted in significant reductions in HBV DNA and RNA levels by 5.88 log IU/mL and 3.13 log IU/mL, respectively. These reductions notably exceeded those achieved with NUCs alone, which led to reductions of 5.21 log IU/mL and 1.43 log IU/mL in HBV DNA and RNA levels [144,145]. Phase 2 trials of JNJ 6379 are currently in progress (NCT04439539 and NCT04667104).

### 5.3. ABI-H0731

ABI-H0731, a derivative of dibenzothiazepinecarboxamide (DBT) belonging to class II CAMs, was evaluated for its anti-HBV efficacy in a phase I clinical trial using a placebo-controlled experimental design. This study included both HBeAg-positive and HBeAg-negative patients with CHB without severe fibrosis. Over a 28-day period, participants were administered daily doses of ABI-H0731 at 100, 200, 300, and 400 mg, or an identical placebo. Treatment at the highest dose was halted due to a maculopapular rash in one participant. Notably, in dose-dependent trials at 100, 200, and 300 mg, reductions of 1.7, 1.9, and 2.9 log IU/mL in HBV DNA levels were observed [146]. Combination therapy studies of ABI-H0731 with ETV in HBeAg-positive CHB patients demonstrated a significant reduction in HBV DNA levels by 5.3 log IU/mL and 2.34 log IU/mL in HBV RNA levels. The reduction of serum HBV RNA may be attributed to CAM, while the reduction of serum HBV DNA may be caused by NUCs (NCT03577171). The trial included a placebo control group. Another investigation combining ABI-H0731 with ETV and either TDF or TAF in patients with CHB has also been completed (NCT03780543). Furthermore, phase 2 studies are currently ongoing to investigate the effectiveness of combining ABI-H0731 with ETV and pegylated IFN-*α* (NCT04781647). Studies are currently underway to assess the combination of ABI-H0731 with various NUCs analogs and JNJ-73763989 (NCT04820686). Unfortunately, no results have yet been reported.

### 5.4. AB-423

The potent SBA capsid inhibitor AB-423 (Arbutus Biopharma, USA) operates through a unique dual mechanism that inhibits HBV core antigen aggregation and pgRNA encapsidation (EC_50_ of 260 nM in vitro). AB-423 is effective against diverse HBV genotypes, including drug-resistant viruses, in cell culture models, without cytotoxicity. The combination of AB-423 with NUCs analogs or interferons enhances its antiviral activity. AB-423 lowers serum HBV DNA levels in mice in a dose-dependent manner. Combining AB-423 with NUCs analogs, RNAi agents, or IFN enhances its antiviral effect. In vivo studies using a mouse model have demonstrated dose-dependent reductions in serum HBV DNA levels following AB-423 treatment [28].

## 6. Conclusions

Therapeutic interventions that can successfully eliminate CHB infection by eradicating cccDNA from infected hepatocytes are currently lacking. Therefore, an alternative objective of emerging therapeutic strategies is to achieve sustained absence of HBsAg for at least 6 months post-treatment. In this context, CAMs have emerged as a promising group of therapeutic compounds. This discussion focused on the chemical structure and antiviral efficacy of the most recently identified sulfamoyl-based carboxamide CAMs, including SBAs, sulfamoyl bicyclic carboxamides, sulfamoyl aliphatic heterocyclic carboxamides, sulfamoyl aromatic heterocyclic carboxamides, cyclic sulfonamides, and non-carboxamide sulfamoyl-based bioactive molecules. Additionally, we focused on the ongoing clinical phase II research involving sulfonyl-based carboxamide CAMs, either as monotherapy or in combination with authorized NUCs or other immunomodulatory agents, to assess their antiviral efficacy.

## Figures and Tables

**Table 1:** Frequently used in vitro cell culture models for HBV infection study.

Cell Culture Models	Pros and Cons of Cell Culture Models			
	Entry	Replication	Secretion	*De Novo* Spread
PHHs	high	high	low	low
HepaRG	low	high	low	no
HuH7	no	moderate	moderate	no
HuH7-NTCP	high	moderate	low	no
HepAD38/HepG2.2.15/HepG2.117	no	high	high	no
HepG2-NTCP	high	high	low	no
HepG2-NTCP sec+	high	high	moderate	moderate
